# Radiation therapy enhances systemic antitumor efficacy in PD-L1 therapy regardless of sequence of radiation in murine osteosarcoma

**DOI:** 10.1371/journal.pone.0271205

**Published:** 2022-07-11

**Authors:** Shohei Katsuki, Yutaka Takahashi, Keisuke Tamari, Kazumasa Minami, Wataru Takenaka, Yoriko Ibuki, Junya Yamamoto, Shotaro Tatekawa, Kazuhiko Hayashi, Yuji Seo, Fumiaki Isohashi, Kazuhiko Ogawa, Masahiko Koizumi

**Affiliations:** 1 Department of Medical Physics and Engineering, Osaka University Graduate School of Medicine, Suita, Osaka, Japan; 2 Department of Radiation Oncology, Osaka University Graduate School of Medicine, Suita, Osaka, Japan; CCAC, UNITED STATES

## Abstract

Recent studies demonstrate that immune checkpoint blockade (ICB) increases the chances of the abscopal effect, an anti-tumor effect outside the radiation field in radiation therapy. However, the optimal sequence between radiation and ICB remains unclear. To investigate the impact of sequence of radiation in anti-PD-L1 antibody (P1) therapy on immune microenvironments and antitumor efficacies in local and abscopal tumors, metastatic LM8 osteosarcoma cells were inoculated into both legs of C3H mice. For irradiation, only one side leg was irradiated at 10 Gy. Then mice were divided into four groups: administrated anti-PD-L1 antibody three times (P1 monotherapy), receiving radiation 3 days prior to P1 therapy (P1+pre-Rad), and receiving concurrent radiation with P1 therapy (P1+conc-Rad). Thereafter, tumor immune microenvironment and tumor volume changes were analyzed in irradiated and unirradiated tumors. The P1+pre-Rad regimen increased the proportion of CD8+ programmed cell death 1 (PD-1)+ granzyme B (GzmB)+ reinvigorated T cells and decreased the proportion of CD8+ PD-1+ GzmB- exhausted T cells than P1+conc-Rad regimen in unirradiated tumors. Combination regimens suppressed tumor growth in irradiated tumors compared with that in P1 monotherapy. In both irradiated and unirradiated tumors, significant tumor growth suppression and prolonged overall survival were observed under both combination treatment regimens compared with P1 monotherapy. However, no distinct differences in unirradiated tumor volume and survival were observed between P1+pre-Rad and P1+conc-Rad groups. These results suggest that local irradiation is necessary to improve systemic treatment efficacy in P1 therapy regardless of sequence of local irradiation.

## Introduction

Immunotherapy is the fourth pillar of cancer therapy [1]. The immune system is a sophisticated biological system that protects the human body from exogenous antigens, such as viruses, and tumors [2]. However, tumors can evade the immune system by promoting immune suppression through the increased expression of immune checkpoint molecules, such as programmed cell death protein-1 (PD-1) on T cells and its ligand (PD-L1) [3–5]. The tumor evasion phase involves loss of antigen presentation and recruitment of immunosuppressive cells [6]. Immune checkpoint blockade, such as anti-PD-L1 (P1) antibody, can overcome this phase and stimulate the immune system into attacking the cancer again [7].

Radiation therapy is a common modality for localized cancers. Preclinical studies using various tumor models have demonstrated that a lethal dose of irradiation to cancer cells induces immunogenic cell death, triggering a strong systemic antitumor immunity [8, 9]. During immunogenic cell death, several mediators are translocated to the cell surface or released [8–12]. For example, calreticulin (CRT), which is an endoplasmic reticulum (ER)-resident protein, functions as an “eat me” signal when translocated to the cell membrane surface by external stimuli [10]. Heat shock proteins, such as heat shock protein 70 (HSP70), which promote the uptake of tumor antigens by dendritic cells (DCs) [11–13], are translocated to the cell surface and released with a tumor associated antigen complex. High mobility group box 1 (HMGB-1) is released into the extracellular milieu and activates DCs through toll-like receptor 4 (TLR4) [9, 14]. These molecules, known as damage associated molecule patterns (DAMPs), induce dendritic cell activation, leading to T-cell activation [8, 9, 14, 15]. Studies have reported that radiation induces the abscopal effect, which regresses tumors outside the radiation field [16–19]. This phenomenon has been found to be an immune-mediated event and includes T-cell activation through DAMPs [9]. Although this is a rare event, immune checkpoint blockade combined with radiation therapy increases the chances of occurrence of the abscopal effect [20–24].

Recently, we reported that compared with P1 and anti-CTLA-4 (C4) therapy, X-ray irradiation combined with P1C4 suppressed tumor growth in both local and distant tumors and prolonged overall survival in osteosarcoma [20]. However, dual immune checkpoint blockade therapy significantly increases medical costs and the risk of immune-related adverse events [3, 25, 26]. Mounting evidence indicates that radiation with concurrent immunotherapy targeting the PD-1/PD-L1 pathway enhanced antitumor efficacy against breast cancer [21, 27], colon carcinoma [21, 28], pancreatic ductal adenocarcinoma [29], glioma [30], renal cell carcinoma [27], and melanoma [31]. However, only a few studies have directly compared the treatment efficacies in local and abscopal tumors between sequential radiation and concurrent radiation in PD-L1 therapy, moreover, the optimal sequence remains unclear particularly for osteosarcoma. Here we show that local irradiation is necessary to enhance the systemic antitumor response in P1 therapy regardless of sequence of local irradiation.

## Materials and methods

### Cell lines and reagents

LM8 murine osteosarcoma and MG63 human osteosarcoma cell lines were purchased from RIKEN (Saitama, Japan) and ATCC (VA, USA), respectively. These were maintained in Dulbecco’s Modified Eagle Medium (DMEM) supplemented with 10% FBS, 5 mM penicillin/streptomycin, and L-glutamine in an incubator at 37.0°C in 5% CO_2_ atmosphere.

### In vitro irradiation

Cells were plated on dishes and maintained overnight in 5% CO_2_ atmosphere and irradiated at 10 Gy using a Gammacell 40 Exactor (Shimazu, Kyoto, Japan). Immediately thereafter, we replaced the cell culture medium and maintained cells in the incubator.

### Ethics statement

Mice were maintained in a pathogen-free area at Osaka University, Suita, Osaka, Japan. All *in vivo* experiments were approved by the Osaka University Institute Animal Use Committee (30-014-005) in accordance with the principles and procedures outlined in the Japanese Act on the Welfare and Management of Animals and Guidelines for the Proper Conduct of Animal Experiments issued by the Scientific Council of Japan. For the survival study, mice were observed daily and humanely sacrificed using CO_2_ gas inhalation when tumor’s longer diameter reached ≧20 mm or they met the following criteria: difficulties in breathing, epistaxis, or rotation motion. Also, we considered the use of buprenorphine when mice were experienced unbearable pain.

### In vivo experiment

Six-week-old C3H/HeNJcl mice were purchased from Nihon-Clea (Tokyo, Japan). LM8 cells (3 × 10^5^ cells in 60 μL PBS) were injected into both legs of mice, as described previously [20]. Treatments with P1 or photon irradiation were initiated when tumor volume exceeded a certain volume (>14 mm^3^). Mice were assigned to five groups, namely untreated (NoTx); P1-administrated (clone 10F.9G2) on days 0, 3, and 6 (P1 only group); irradiated on one side of the tumor on day 0 (Rad only group); sequentially irradiated with photon beams to the tumor on one leg on day 0 followed by P1 administration on days 3, 6, and 9 (P1+pre-Rad group); and concurrently irradiated with photon beams on day 3 with P1 administration on days 0, 3, and 6 (P1+conc-Rad group).

For mice in the P1 only, P1+pre-Rad, and P1+conc-Rad groups, P1 was administered through intraperitoneal (i.p.) injection at 150 μg in 100 μL PBS per dose. To retain mice during irradiation 40 mg/kg pentobarbital sodium was administered through i.p. injection and fixed on an in-house jig. The legs on one side of the mouse were irradiated at 10 Gy by using the orthovoltage X-ray irradiator [20] or Gammacell 40 Exactor. We ensured that photon beams were only delivered to legs on one side of leg while the rest of body was enough shielded by lead blocks.

Tumor volume was calculated using the following formula: “(Length) × (Width)^2^ × 0.52”. Length and width were measured at least every 3 days and the measured tumor volume data was binned every 3 days.

### Flow cytometry

*In vitro* flow cytometry was performed by staining irradiated or unirradiated cells with calreticulin-FITC (Bioss Antibodies, MA, USA, clone Ag04284950) diluted with FACS buffer (PBS supplemented with 2% FBS) at a ratio of 1:100 for 1 h on ice and HSP70-FITC (clone 1H11) (StressMarq Biosciences, BC, CA) diluted with FACS buffer at a ratio of 1:250 for 1 h on ice.

For *in vivo* tumor immune microenvironment analyses, tumors harvested from NoTx, P1 only, Rad only, P1+pre-Rad, and P1+conc-Rad groups were minced in FACS buffer. Then, a single cell suspension was prepared using 0.5 mg/mL collagenase IV (Sigma Aldrich, MO, USA) with 200 μg/mL DNAse (Sigma Aldrich, MO, USA) as described previously [20].

The single cell suspension, containing 1 × 10^6^ cells, was incubated with anti-mouse CD16/32 antibody (BioLegend, CA, USA) for 10 min at room temperature for Fc blocking and then with CD8-APC (eBioscience, clone 53–6.7), CD4-APC (eBioscience, clone RM4-5), CD11c-PE (eBioscience, clone N418), CD103-APC (eBioscience, clone 2E7), CD45-FITC (eBioscience, clone 30-F11), and CD279 (PD-1)-FITC (eBioscience, clone J43). Each antibody was diluted in FACS buffer at a ratio of 1:80, except CD45 and PD-1, which was diluted at a ratio of 1:100, and was added to react for 30 min on ice. For GzmB staining, fixation, followed by permeabilization was performed using the FoxP3 staining kit (eBioscience) according to the manufacturer’s instructions. Then, the GzmB-PE antibody (eBioscience, clone NGZB) was incubated at a ratio of 1:80 for 30 min on ice. After washing, stained cells were analyzed using FACS Verse^TM^ (BD, NJ, USA). Data analysis was performed using FlowJo® ver.10.4.2 (BD).

### Enzyme-linked immunosorbent assay

The release of HMGB-1 into the culture supernatant was measured using *enzyme-linked* immunosorbent assay kits (Arigobio, Taiwan). All procedures were performed according to the manufacturer’s instructions.

### Statistics

To compare CRT and HSP70 expression and HMGB-1 released into cell culture at designated times and proportions of immune cells in tumors between P1+pre-Rad and P1+conc-Rad groups, a two-tailed Student’s *t*-test was performed. Time-dependent changes in CRT and HSP70 expression were compared using Tukey’s honestly significant difference test. The proportion of immune cells between NoTx, P1 only, and Rad only groups were compared using Dunnett’s test. Differences in tumor volumes between NoTx, P1 only and Rad only groups were evaluated using Dunnett’s test. Differences in tumor volume between P1 only, P1+pre-Rad and P1+conc-Rad groups were compared using Tukey’s honestly significant difference test. Differences in tumor volume between combination groups (P1+pre-Rad and P1+conc-Rad groups) and P1 only group were evaluated using a two-tailed Student’s *t*-test. The Kaplan–Meier method was used for survival analysis and differences were compared using the log-rank test. *P*-values were adjusted by the method of Bonferroni for multiple comparison.

## Results

### DAMPs expression and release by X-ray irradiation in vitro

**Fig 1:**
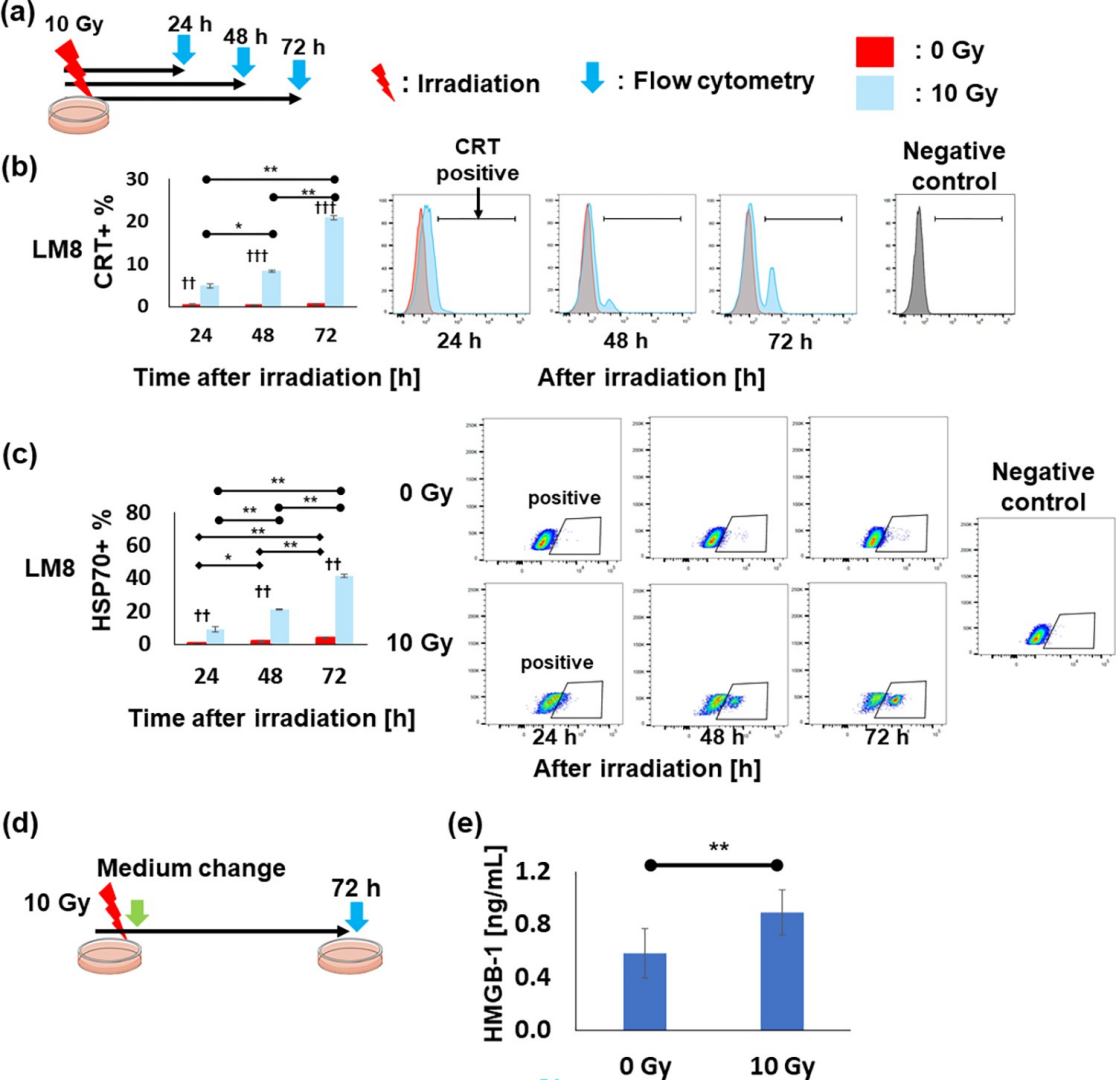
DAMPs expression and release by 10 Gy irradiation at various time points. (a) A scheme of CRT and HSP70 expression analysis. (b) Proportions of translocated CRT on LM8 cell. Panels on the right are representative histograms showing the fluorescent intensity of CRT at each time point. (c) Proportions of HSP70 expressed on LM8 and MG63 cells. Panels on the right are representative histograms showing the fluorescent intensity of HSP70 at each time point. (d) An experimental scheme of HMGB-1 measurement in cell culture supernatants. (e) HMGB-1 concentration in cell culture supernatants of LM8 72 h after 10 Gy irradiation. *p < 0.05, **p < 0.01, ***p < 0.001, ****p < 0.0001; Data represent the mean ± SEM. Significance was evaluated using a two-tailed Student’s *t*-test. For multiple comparisons, *p*-values were adjusted using the Bonferroni correction. Abbreviations; DAMPs: damage associated molecular patterns, CRT: calreticulin, HSP70: heat shock protein 70.

To investigate the kinetics of DAMPs after irradiation, we irradiated LM8 murine osteosarcoma and MG63 human osteosarcoma cell lines at 10 Gy *in vitro* (Fig 1A). Flow cytometric analysis revealed that the percentage of translocated CRT on the irradiated cell surface significantly increased in a time dependent manner (Fig 1B). In LM8 murine osteosarcoma cells, 4.9% and 8.3% CRT positive cells were observed at 24 h and 48 h following irradiation, respectively. A similar trend was observed in MG63 cells (S1 Fig).

HSP70 expression on the cell surface was also increased in a time dependent manner on LM8 and MG63 cells (Fig 1C and S1 Fig). In particular, HSP70 expression on LM8 cells significantly increased to 41.5% at 72 h following 10 Gy irradiation, compared to 3.7% in unirradiated cells (*p* < 0.001).

We next examined HMGB-1 released into the culture supernatant 72 h following 10 Gy irradiation (Fig 1D). The concentration of HMGB-1 was 0.90 ng/mL after 10 Gy irradiation, which was 55% higher than that of unirradiated cells (Fig 1E). Also, S2 Fig shows the dot plots of flow cytometric results,

### The effect of radiation on the tumor immune microenvironment

**Fig 2:**
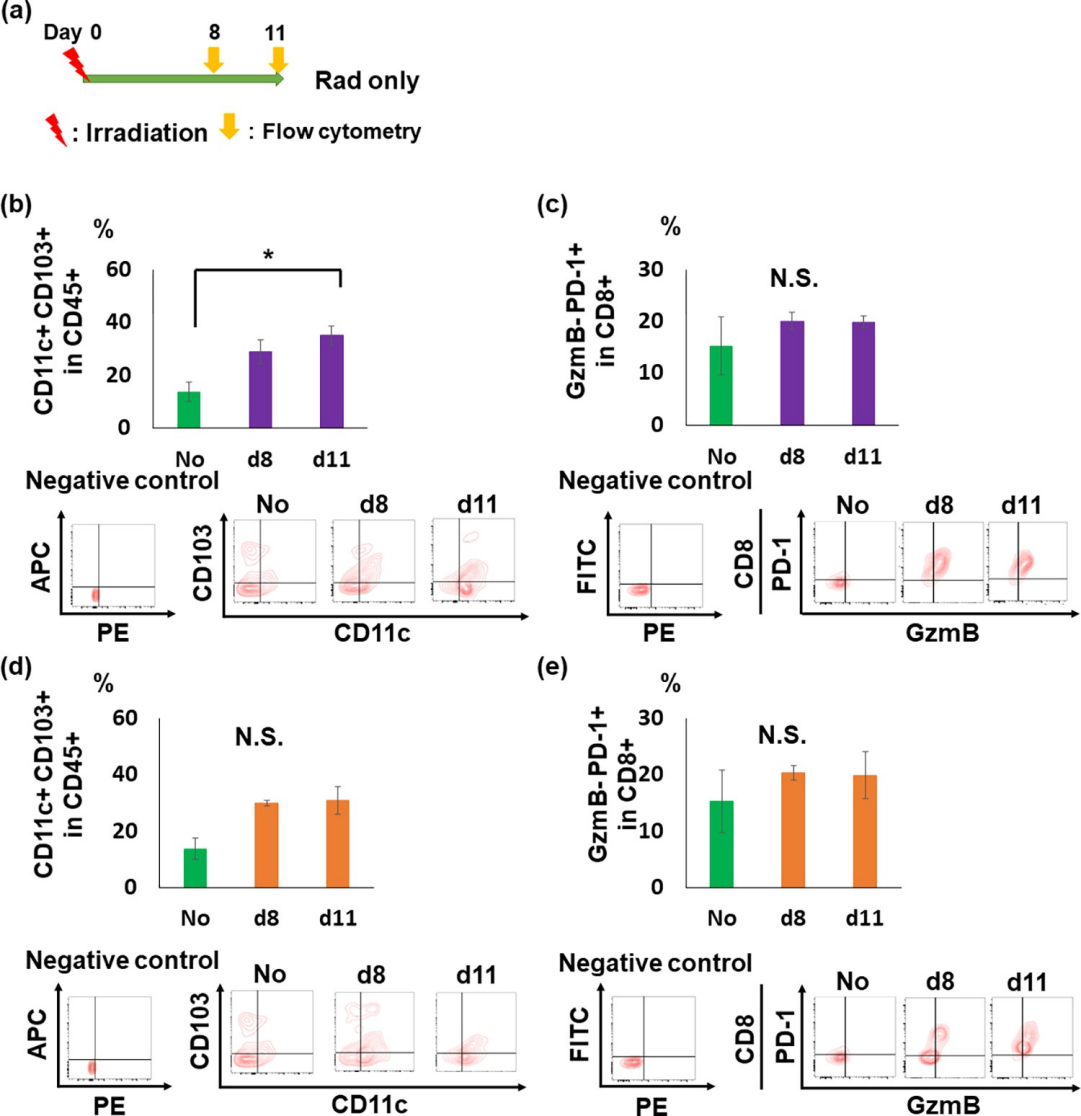
Tumor immune microenvironment change in IR and unIR tumors by local irradiation. (a) An experimental scheme. (b, c) Proportions of activated (CD45+ CD11c+ CD103+) dendritic cells (b) and exhausted (CD8+ GzmB- PD-1+) T cells (c) in IR tumors (n = 6). (d, e) Proportions of activated (CD45+ CD11c+ CD103+) dendritic cells (d), cytotoxic (CD8+ GzmB+) T cells (e), and exhausted (CD8+ GzmB- PD-1+) T cells (g) in unIR tumors (n = 6). Data are expressed as mean ± SEM. Data in NoTx (n = 5) group were shared both in IR (b, c) and unIR (d, e) graphs, because the same materials and procedures were used. Representative contour plots are shown under corresponding graphs. *p < 0.05. Abbreviations; No: No treatment group, IR: irradiated, unIR: unirradiated, GzmB: granzyme B.

We evaluated changes in the immune microenvironment in irradiated (IR) and unirradiated (unIR) tumors 8 days and 11 days after 10 Gy irradiation *in vivo* (Fig 2A). Recent reports demonstrated that CD103+ DCs contributes to T-cell activation [32]. Flow cytometric analysis revealed that activated DCs (CD45+ CD11c+ CD103+) in IR tumors increased by 17.1% 11 days after irradiation, compared with those in the NoTx group (*p* = 0.0315, Fig 2B). Although activated DCs increased after irradiation, the proportion of exhausted T cells (CD8+ GzmB- PD-1+) did not change after local irradiation (Fig 2C).

In contrast to those in IR tumors, local radiation did not affect the proportions of activated DCs, and exhausted T cells in unirradiated tumors (Fig 2D and 2E and S3 Fig). These results suggest that radiation can alter the tumor microenvironment only in IR tumors.

### Radiation prior to anti-PD-L1 antibody improved the tumor immune microenvironment

**Fig 3:**
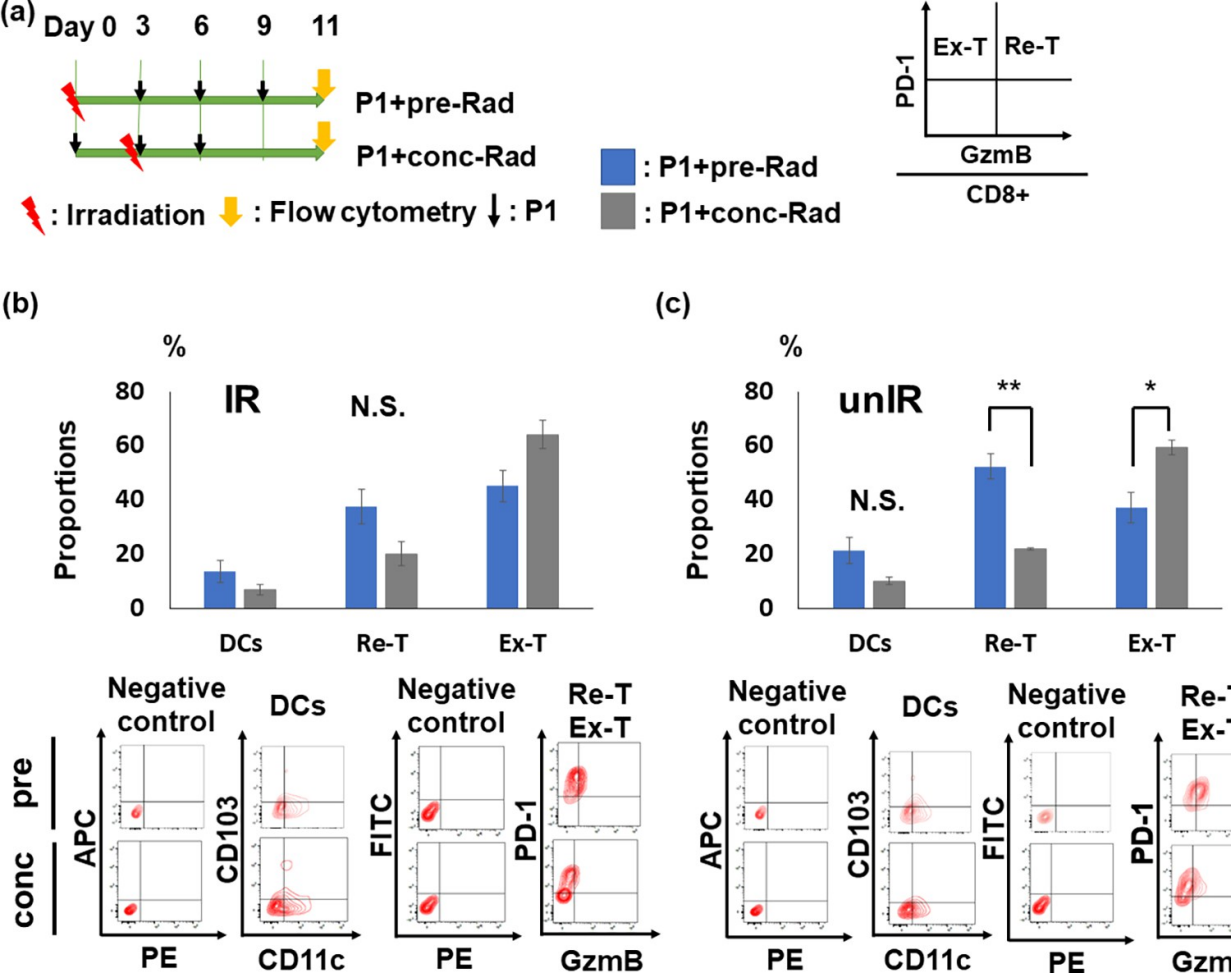
Comparison of the tumor immune microenvironment due to different sequences of radiation in P1 therapy. (a) A treatment scheme for P1+pre-Rad (n = 7) and P1+conc-Rad (n = 4) groups. (b and c) Proportions of activated DCs (DCs), reinvigorated T cells (Re-T), and exhausted T cells (Ex-T) in IR (b) and unIR tumors (c). Data are expressed as mean ± SEM. Representative contour plots are shown under corresponding graphs. *p < 0.05, **p < 0.01. Abbreviations; P1: anti-PD-L1 antibody, IR: irradiated, unIR: unirradiated.

To evaluate whether addition of P1 to radiation at different sequence alters the tumor immune microenvironment, we examined the proportions of activated DCs, cytotoxic T cells, and exhausted T cells in irradiated (IR) and unirradiated (unIR) tumors 11 days after the initial treatment (Fig 3A).

Our results revealed that proportions of activated DCs, reinvigorated T cells, and exhausted T cells were not altered in IR tumors (Fig 3B; Re-T and Ex-T). By contrast, in unIR tumors, a significant increase in reinvigorated T cells and decrease in exhausted T cells was observed in the P1+pre-Rad group compared with P1+conc-Rad group (*p* = 0.0042 and 0.0482, respectively; Fig 3C; Re-T and Ex-T and S4 Fig). These results indicated that neoadjuvant but not concurrent radiation with P1 therapy improved the tumor immune microenvironment at distant tumors.

### The effect of the treatment sequence between radiation and P1 therapy on the local and systemic antitumor efficacies

**Fig 4:**
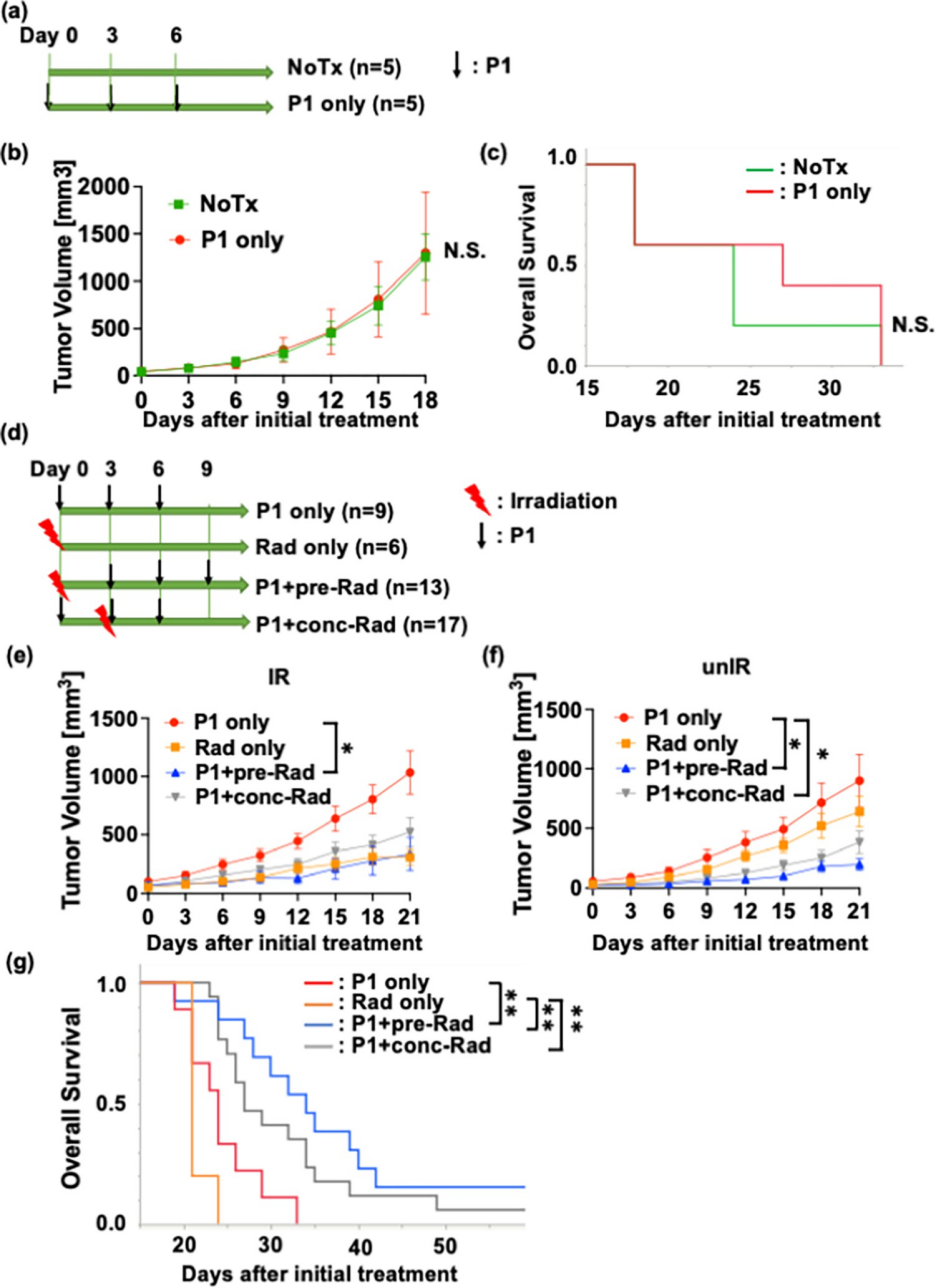
Comparison of treatment efficacies. (a) A treatment scheme of NoTx (n = 5), P1 only (n = 5) groups. (b and c) Tumor volume change (b) and overall survival (c) in NoTx (green) and P1 only (red) groups. Data are presented as mean ± SEM. (d) A treatment scheme in P1 only (n = 9), Rad only (n = 6), P1+pre-Rad (n = 13), and P1+conc-Rad (n = 17) groups. (e and f) Tumor volume change in IR (e) and unIR tumors (f). Data are presented as mean ± SEM. (g) Overall survival in P1 only, Rad only, P1+pre-Rad, and P1+conc-Rad groups. *p < 0.05, **p < 0.01. Abbreviations; NoTx: no treatment, P1; anti-PD-L1 antibody, Rad only: radiation only, IR: irradiated, unIR: unirradiated.

First, we investigated therapeutic efficacy with P1 monotherapy (Fig 4A). The tumor volume changes and overall survival in P1 only and NoTx groups were almost identical (Fig 4B and 4C). Therefore, we set P1 only group as a control group in the following experiments.

To evaluate the difference in the therapeutic efficacy by sequence between radiation and P1, mice were divided into three groups, namely P1 only, Rad only, P1+pre-Rad, and P1+conc-Rad groups as described in Fig 4D. Analysis for IR tumor volume changes revealed that radiation monotherapy suppressed tumor growth by 61.1% (*p* = 0.0783 at day 18) compared with P1 monotherapy (Fig 4E) but did not reach statistical significance. In contrast, P1 therapy combined with sequential and concurrent radiation suppressed tumor growth by 65.4% (*p* = 0.0127) and 48.5% (*p* = 0.0734) 18 days after the initial treatment, respectively, compared with P1 monotherapy (Fig 4E). No differences in IR tumor volume change were observed between Rad only, P1+pre-Rad, and P1+conc-Rad groups.

Moreover, tumor growth was not suppressed by radiation monotherapy for unIR tumor volume (Fig 4E). In contrast, mice in the P1+pre-Rad, and P1+conc-Rad groups were experienced significant tumor growth delay by 74.9% (*p* = 0.0021) and 64.7% (*p* = 0.0057) 18 days after the initial treatment, respectively, compared with P1 only group (Fig 4F). These results suggest that combination of P1 and radiation is required to enhance local and abscopal responses regardless of sequence. Consistent with these findings, compared with P1 monotherapy, P1+pre-Rad and P1+conc-rad treatments prolonged overall survival. Median survival time was 24, 21, 34, and 27 days in P1 only, Rad only (*p* = 0.94), P1+pre-Rad (*p* = 0.0085), and P1+conc-Rad (*p* = 0.0600) groups, respectively (Fig 4G and S6 Fig). Furthermore, compared with Rad only group, both P1+Pre-Rad and P1+conc-Rad significantly prolonged overall survival (*p* = 0.0043 and *p* = 0.002, respectively). Taken together these results suggest that addition of radiation to P1 therapy is necessary to enhance systemic antitumor immune response and improve survival regardless of sequence of radiation.

## Discussion

Osteosarcoma is one of the most common primary bone tumors in children and adolescents [20, 33]. Although chemotherapy is the primary choice for treating distant metastasis, its efficacy remains limited [33]. Immune checkpoint blockade therapy has attracted attention but P1 monotherapy was reported to have limited efficacy in various cancers in a clinical trial [34]. We selected the LM8 mice model, which easily induces distant metastases [35], enabling us to evaluate the abscopal effect as well as the local effects.

Our data also showed that no therapeutic gain was achieved by P1 monotherapy (Fig 4B and 4C). Furthermore, radiation monotherapy at 10 Gy in a single fraction to one side of leg was large to exert substantial antitumor effect only at irradiated tumors, but not at abscopal tumors, indicating that radiation monotherapy is not enough to induce systemic antitumor effects.

To improve the antitumor response by immune checkpoint blockade, Victor et al. demonstrated that X-ray irradiation combined with dual immune checkpoint blockade (P1C4) enhanced the treatment efficacy for both local and distant tumors in various cancers, including C4 resistant melanoma [34]. More recently, our group reported that X-ray or carbon ion beam irradiation combined with dual immune checkpoint blockade induced systemic tumor growth suppression and prolonged overall survival [20, 22]. However, dual immune checkpoint therapy significantly increases the risk of immune related adverse events for patients with non-small cell lung carcinoma, melanoma, and renal cell carcinoma presenting with distant metastasis [3, 25, 26]. Therefore, we explored whether the therapeutic responses achieved by radiation therapy combined with P1 therapy are dependent on the sequence of radiation therapy.

Studies have reported that X-ray irradiation and concurrent use of P1 successfully enhanced local control in murine breast cancer [36], murine colon carcinoma [28], and murine pancreatic ductal adenocarcinoma models [29]. Although Daniel A. et al. reported the study investigating the impact of treatment sequence on the therapeutic efficacy using metastatic mouse model with melanoma [37], this is the first report that directly compared antitumor efficacies between concurrent and sequential use of radiation in P1 therapy for osteosarcoma. Moreover, correlation of the time interval between radiation and P1 administration with the abscopal effect remains unclear, especially for osteosarcoma. To the best of our knowledge, this is the first study to directly compare therapeutic efficacies using different sequences of radiation and P1 and to demonstrate the need for adding radiation to P1 monotherapy for osteosarcoma.

The mechanism underlying the abscopal effect has been recently unveiled. Induction of the danger signal following tumor irradiation was associated with the abscopal effect [15, 21]. Several studies reported that CRT and HSP70 translocation on the cell surface and HMGB-1 release increased 72 h following 10 Gy irradiation [8, 38], which induced T-cell activation by increasing the ability of antigen presentation by DCs [9, 14, 15]. Similarly, our study revealed that these phenomena increased in a time-dependent manner and lasted until 72 h after 10 Gy irradiation. Therefore, we hypothesized that P1 administration 72 h following X-ray irradiation may more effectively induce cytotoxic T-cell proliferation through DC activation, thereby suppressing the growth of both IR and unIR tumors. The analysis of the IR tumor immune microenvironment revealed that irradiation partially altered the immune microenvironment toward favorable conditions to induce the antitumor response as the DC activation was induced by local irradiation.

Furthermore, X-ray irradiation 3 days prior to P1 administration (P1+pre-Rad) induced more favorable tumor microenvironment at 11 days after the initial treatment than concurrent treatment by radiation with P1 therapy (P1+conc-Rad), including increased reinvigorated T cells with lower exhausted T cells. Although the P1+pre-Rad regimen improved the tumor immune microenvironment, unIR tumor growth were similarly suppressed by both P1+pre-Rad and P1+conc-Rad regimens, suggesting that addition of radiation to P1 therapy is crucial for achieving systemic tumor response regardless of sequence of radiation in P1 therapy. Further analysis revealed that both P1+pre-Rad and P1+conc-Rad regimens successfully suppressed tumor growth not only in local, but also distant tumors. However, only 2 (15%) and 1 (5.9%) mice in P1+pre-Rad and P1+conc-Rad groups, respectively, survived for more than 50 days after the initial treatment, whereas our previous studies demonstrated that 3 of 7 (42.9%) mice in P1C4 with radiation therapy at 10 Gy [20] and more than 30% in C4 with radiation at 16 Gy or 8 Gy × 3 fraction groups [39] survived for more than 50 days after the initial treatment. Therefore, further improvement in therapeutic efficacies may be achieved by using escalated dose to a local tumor.

Azad et al. [29] reported that P1 administration 7 days post radiation failed to control primary tumor growth in pancreatic ductal adenocarcinoma. In those studies, P1 administered 3 days after radiation led to a better outcome, suggesting the presence of an optimal window between radiation, which triggers danger signals and activation of antitumor immunity, and P1 administration.

In conclusion, P1+pre-Rad altered the immune microenvironment in unIR tumors toward more favorable conditions compared with P1+conc-Rad. By contrast, similar treatment efficacies in unIR tumor were obtained using both regimens, leading to prolonged overall survival. Thus, regardless of sequence of radiation, local radiation therapy is indispensable for increasing the abscopal effect and improving survival in P1 therapy for osteosarcoma, indicating the flexibility of radiation timing when combined with P1 treatment in a certain range.

## Supporting information

S1 Fig DAMPs expression on MG63 cell. (a) Proportions of translocated CRT. Right panels are representative histograms showing the intensity at each time point. (b) Proportions of HSP70. Right panels are representative pseudocolor map showing fluorescent intensity of HSP70 at each time point after 10 Gy irradiation. *p < 0.05, **p < 0.01, ***p < 0.001, ****p < 0.0001; Data represent the mean ± SEM. Significance was evaluated using a two-tailed Student’s *t*-test. For multiple comparisons, *p*-values were adjusted using the Bonferroni correction. Abbreviations; DAMPs: damage associated molecular patterns, CRT: calreticulin, HSP70: heat shock protein 70.
(TIF)

S2 Fig Individual data of the proportions of CRT and HSP70 expression and release of HMGB-1. (a and b) The expression of CRT and HSP70 on LM8 cells. (c and d) The expression of CRT and HSP70 on MG63 cells. (e) The release of HMGB-1. Abbreviations; CRT: calreticulin, HSP70: heat shock protein 70. HMGB-1: high mobility group box 1.
(TIF)

S3 Fig Individual data of immune cells in tumor microenvironment. (a and c) Proportions of DC and Ex-T in IR tumors. (b and d) Proportions of DC and Ex-T in unIR tumors. Abbreviation; NoTx: no treatment.
(TIF)

S4 Fig Individual data of immune cells in tumor microenvironment. (a, c, and e) Proportions of immune cells in IR tumors. (b, d, and f) Proportions of immune cells in unIR tumors. Abbreviations; pre: P1+pre-Rad, conc: P1+conc-Rad.
(TIF)

S5 Fig Tumor volume change of individual mouse in the four groups. (a) Tumor growth curve are exhibited in green (NoTx) and red (P1only).
(TIF)

S6 Fig Tumor volume change of individual mouse in the groups. (a and d) The larger tumor growth (a) and the other (e) of the two in P1only group are exhibited individually. (b, c, d, e, f, g, and h) The tumor growth in IR tumor (solid line) (b, c, and d) and unIR tumor (dashed line) (f, g, and h) are exhibited in yellow (Rad only), blue (P1+pre-Rad), and gray (P1+conc-Rad).
(TIF)
