# Supplementary figures and images for: Radiation therapy enhances systemic antitumor efficacy in PD-L1 therapy regardless of sequence of radiation in murine osteosarcoma
Source: PLoS One. 2022 Jul 11;17(7):e0271205. doi: 10.1371/journal.pone.0271205 (PMC9273087; doi:10.1371/journal.pone.0271205)

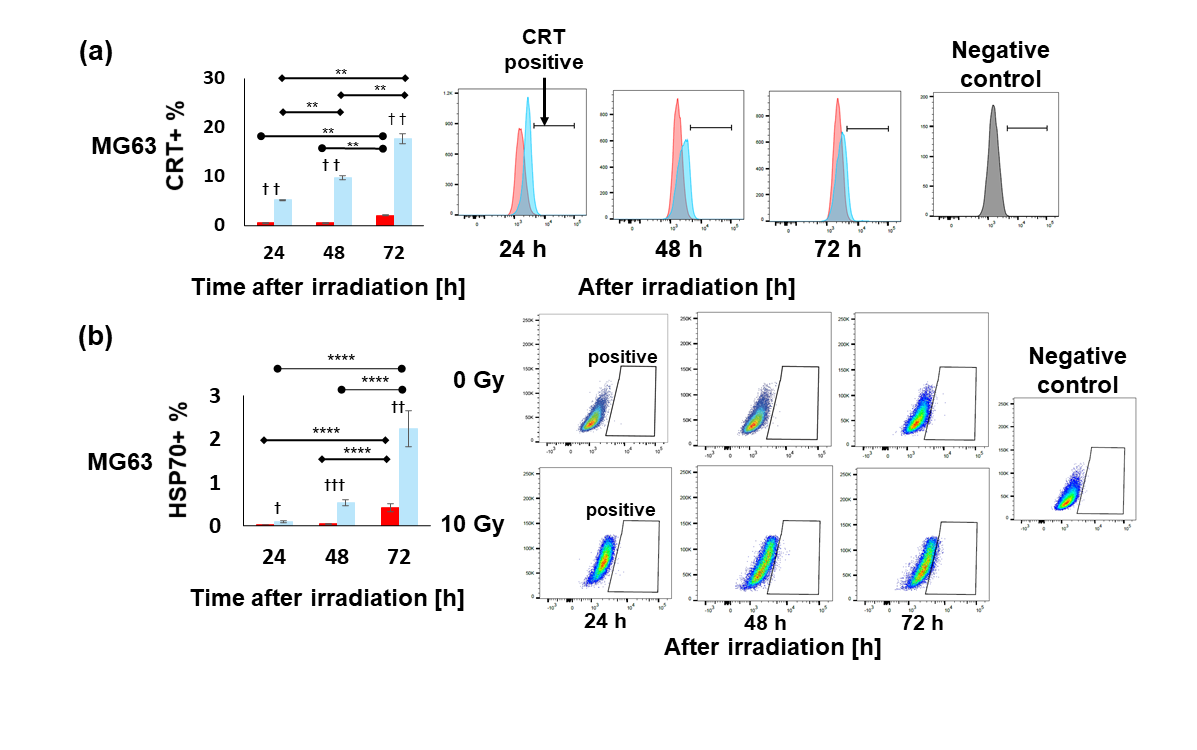

Supplement: S1 Fig — (a) Proportions of translocated CRT. Right panels are representative histograms showing the intensity at each time point. (b) Proportions of HSP70. Right panels are representative pseudocolor map showing fluorescent intensity of HSP70 at each time point after 10 Gy irradiation. *p < 0.05, **p < 0.01, ***p < 0.001, ****p < 0.0001; Data represent the mean ± SEM. Significance was evaluated using a two-tailed Student’s t-test. For multiple comparisons, p-values were adjusted using the Bonferroni correction. Abbreviations; DAMPs: damage associated molecular patterns, CRT: calreticulin, HSP70: heat shock protein 70. (TIF) [file pone.0271205.s001.tif]

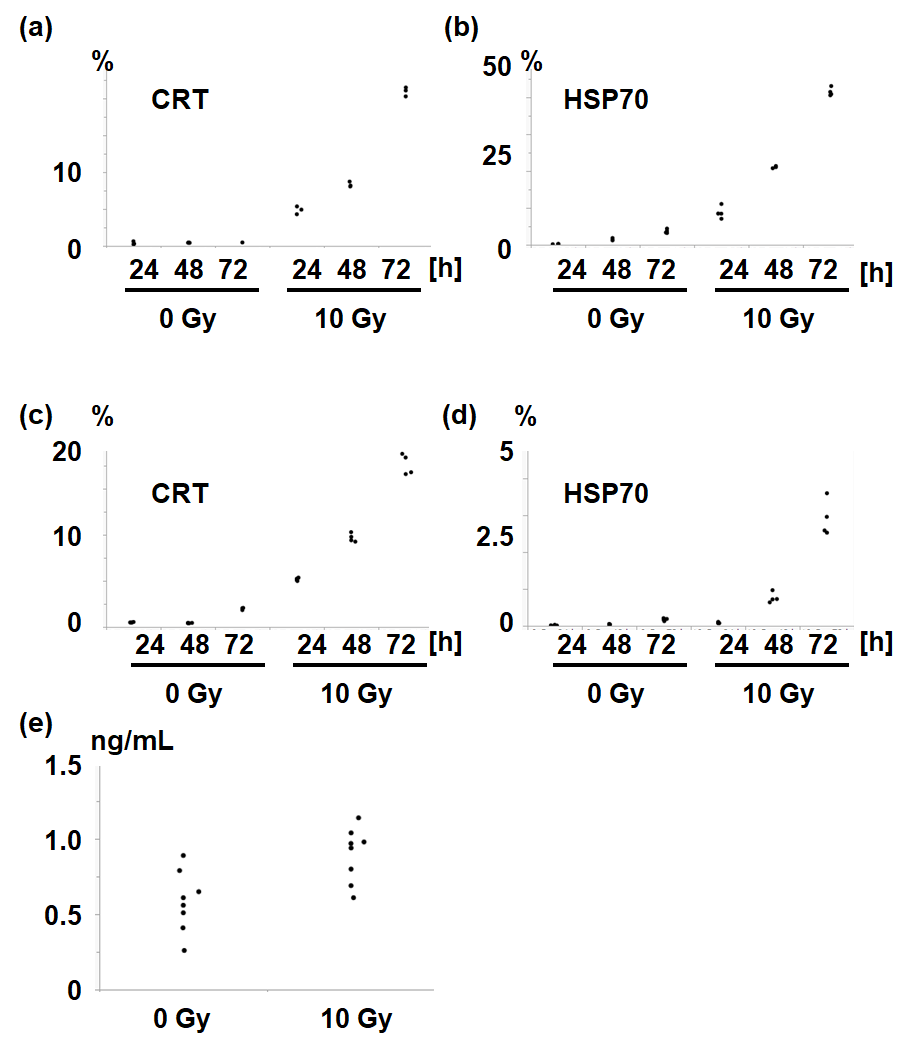

Supplement: S2 Fig — (a and b) The expression of CRT and HSP70 on LM8 cells. (c and d) The expression of CRT and HSP70 on MG63 cells. (e) The release of HMGB-1. Abbreviations; CRT: calreticulin, HSP70: heat shock protein 70. HMGB-1: high mobility group box 1. (TIF) [file pone.0271205.s002.tif]

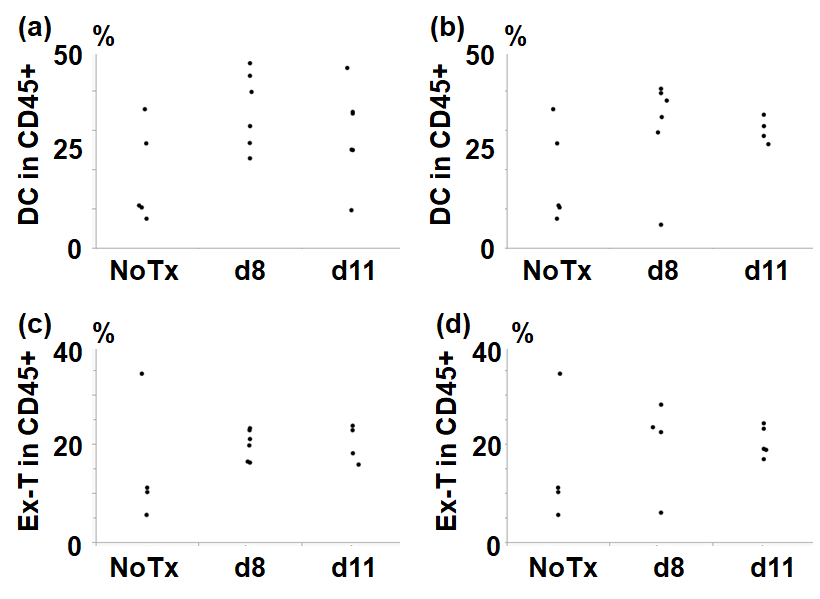

Supplement: S3 Fig — (a and c) Proportions of DC and Ex-T in IR tumors. (b and d) Proportions of DC and Ex-T in unIR tumors. Abbreviation; NoTx: no treatment. (TIF) [file pone.0271205.s003.tif]

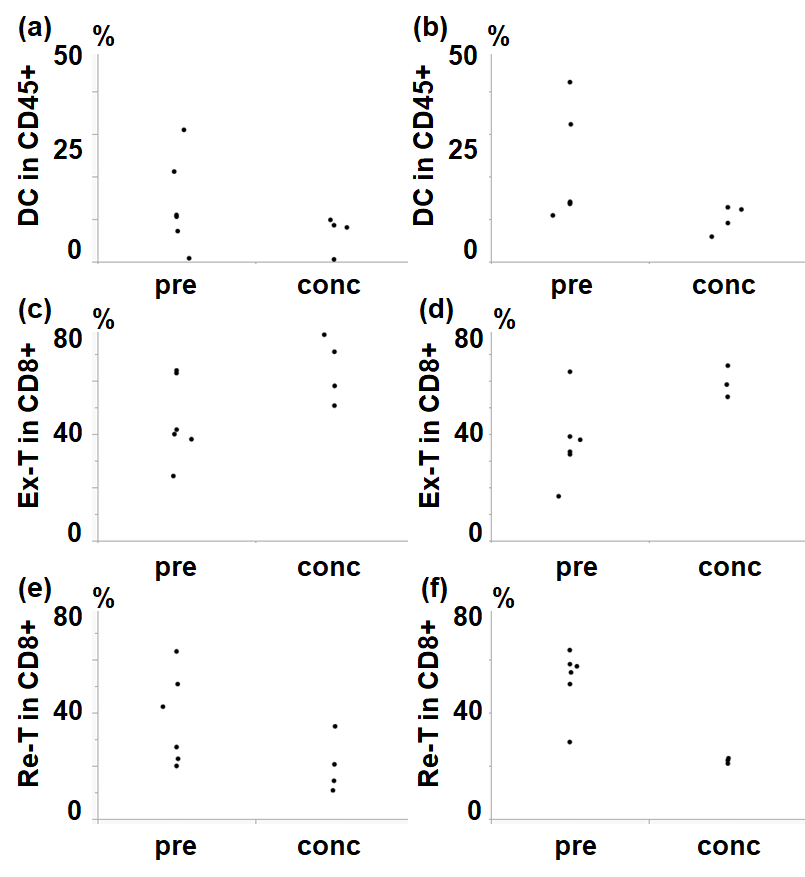

Supplement: S4 Fig — (a, c, and e) Proportions of immune cells in IR tumors. (b, d, and f) Proportions of immune cells in unIR tumors. Abbreviations; pre: P1+pre-Rad, conc: P1+conc-Rad. (TIF) [file pone.0271205.s004.tif]

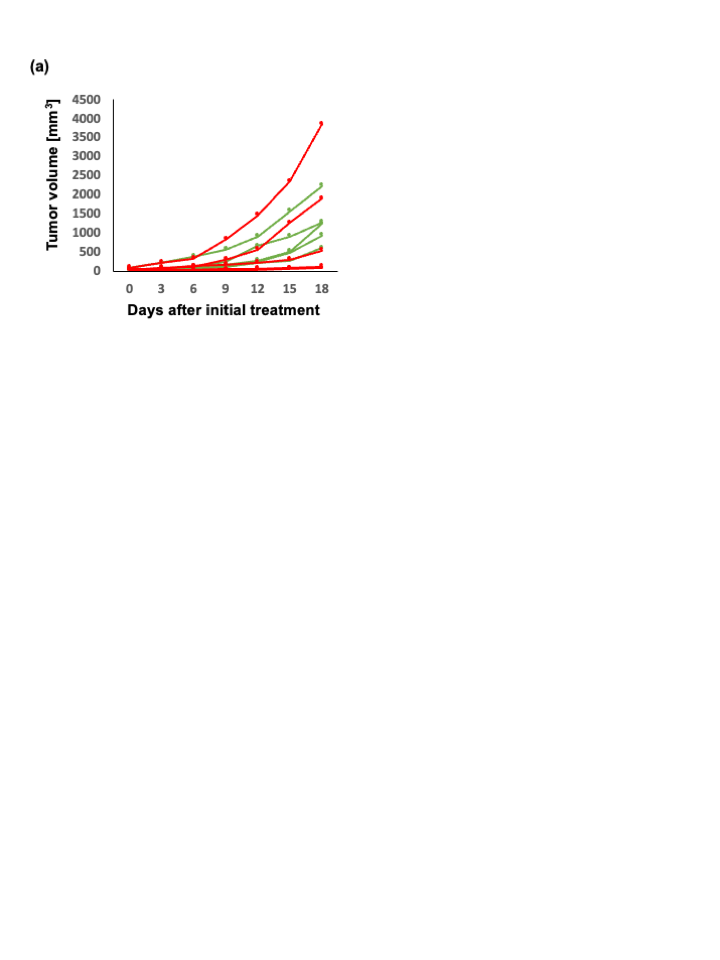

Supplement: S5 Fig — (a) Tumor growth curve are exhibited in green (NoTx) and red (P1only). (TIF) [file pone.0271205.s005.tif]

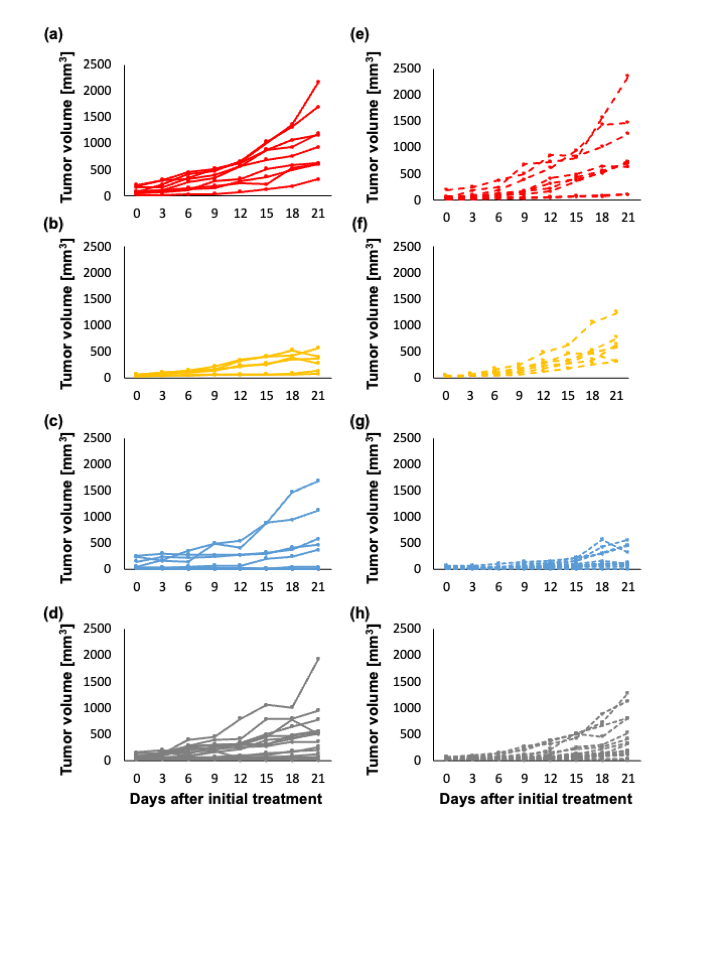

Supplement: S6 Fig — (a and d) The larger tumor growth (a) and the other (e) of the two in P1only group are exhibited individually. (b, c, d, e, f, g, and h) The tumor growth in IR tumor (solid line) (b, c, and d) and unIR tumor (dashed line) (f, g, and h) are exhibited in yellow (Rad only), blue (P1+pre-Rad), and gray (P1+conc-Rad). (TIF) [file pone.0271205.s006.tif]
